# Nocturnal blood pressure rise as a predictor of cognitive impairment among the elderly: a retrospective cohort study

**DOI:** 10.1186/s12877-021-02406-4

**Published:** 2021-08-11

**Authors:** Yunli Xing, Ying Sun, Shan Wang, Feng Feng, Deqiang Zhang, Hongwei Li

**Affiliations:** 1 Department of Geriatrics, Beijing Friendship Hospital, Capital Medical University, Beijing, 100050 PR China; 2 Department of Cardiology, Beijing Friendship Hospital, Capital Medical University, No.95, Yongan Road, Xicheng District, Beijing, 100050 PR China

**Keywords:** Ambulatory blood pressure monitoring, Blood pressure, Nocturnal blood pressure rise, Cognitive impairment

## Abstract

**Background:**

This study investigated the different blood pressure patterns that were evaluated by ambulatory blood pressure monitoring (ABPM) among elderly patients and explored the effect of pressure patterns on cognitive impairment and mortality.

**Methods:**

A total of 305 elderly participants aged ≥65 years were divided into the cognitive impairment group (CI, *n* = 130) and the non-cognitive impairment group (NCI, *n* = 175) according to the MMSE score. All participants underwent ABPM to evaluate possible hypertensive disorder and cerebral MRI for the evaluation of cerebral small vessel disease. Follow-up was performed by telephone or medical records. The primary outcome was all-cause mortality. Secondary endpoints were major adverse cardiac and cerebrovascular events (MACCE).

**Results:**

Among 305 participants, 130 (42.6%) were identified with cognitive impairment (CI), with average systolic blood pressure (BP) of 127 mmHg and diastolic BP of 66 mmHg. According to ABPM, only 13.1% had a dipper pattern, 45.6% had a nocturnal BP rise, while 41.3% had a non-dipper pattern. Compared with NCI patients, the CI group had significantly higher night-time systolic BP (130.0 ± 18.2 vs. 123.9 ± 15.1, *p* = 0.011), and more participants had nocturnal BP rise (52.3% vs. 40.6%, *p* = 0.042). Nocturnal BP rise was associated with greater white matter hyperintensities (WMH) (*p* = 0.013). After 2.03 years of follow-up, there were 35 all-cause deaths and 33 cases of major adverse cardiac and cerebrovascular events (MACCE). CI was independently associated with all-cause mortality during long-term observation (*p* < 0.01). Nocturnal BP rise had no significant predictive ability for all-cause mortality in elderly patients (*p* = 0.178).

**Conclusions:**

Nocturnal BP rise contributed to greater cognitive impairment in elderly patients. Not nocturnal BP rise, but CI could significantly increase all-cause mortality. Controlling BP based on ABPM is critical for preventing the progression of cognitive dysfunction.

## Background

Dementia affects approximately 50 million people worldwide and is expected to increase by nearly 9.9 million new cases each year. While Alzheimer’s disease is the leading cause of cognitive impairment (CI), vascular dementia is the second leading cause, with no effective therapies [1]. Cerebral small vessel disease is the most common pathology underlying vascular dementia, including lacunar infarcts (LCI) or white matter hyperintensities (WMH) [2]. Hypertension, which can affect brain structure and function, is known to be associated with CI. It is the major vascular risk factor for CI [3] . The associations between blood pressure (BP) and brain health are complex and are dependent on many factors such as age, hypertension chronicity, and hypertension variation. The elevated BP in midlife, particularly untreated hypertension, increases the risk for CI. Previous studies have either failed to find any association between hypertension and CI in the eighth, ninth and tenth decade of life or have reported high BP as having protective effect against CI [3].

Twenty-four-hour ambulatory BP monitoring (ABPM) offers much more information on variability and circadian BP pattern compared to office BP measurements and is superior in predicting future cardiovascular events [4]. Although the relationship between blood pressure variation (BPV) and CI has been addressed to some extent, previous studies have yielded conflicting results. McDonald and colleagues reported that daytime systolic variability was independently associated with greater cognitive decline in total CAMCOG and MMSE scores over a 5-year follow-up [5]. Kanemaru et al reported that increased short-term variability of daytime BP and high night-time systolic BP was associated with cognitive impairment as assessed by the Raven’s Coloured Progressive Matrices Test (RCPM) [6]. Paganini-Hill et al assessed the patients aged 90+ years and found that mean nocturnal dips significantly differed between cognitively normal and impaired individuals, with cognitively normal participants having on average greater nocturnal dips. Nocturnal dips were also related to performance on select cognitive test scores [7, 8]. The different study designs, measures of cognition, and population characteristics may explain this inconsistency.

Previous studies have reported on a cognitive impairment-mortality relationship and indicated that baseline cognitive impairment increases the risk of all-cause mortality [9, 10]. However, they have rarely studied the impacts of BPV on the association between cognitive impairment and mortality.

The present study assessed the relationship between BPV and CI in elderly patients with different levels of cognitive function assessed by MMSE and investigated the association of cognitive impairment on mortality.

## Methods

### Participants

**Fig. 1:**
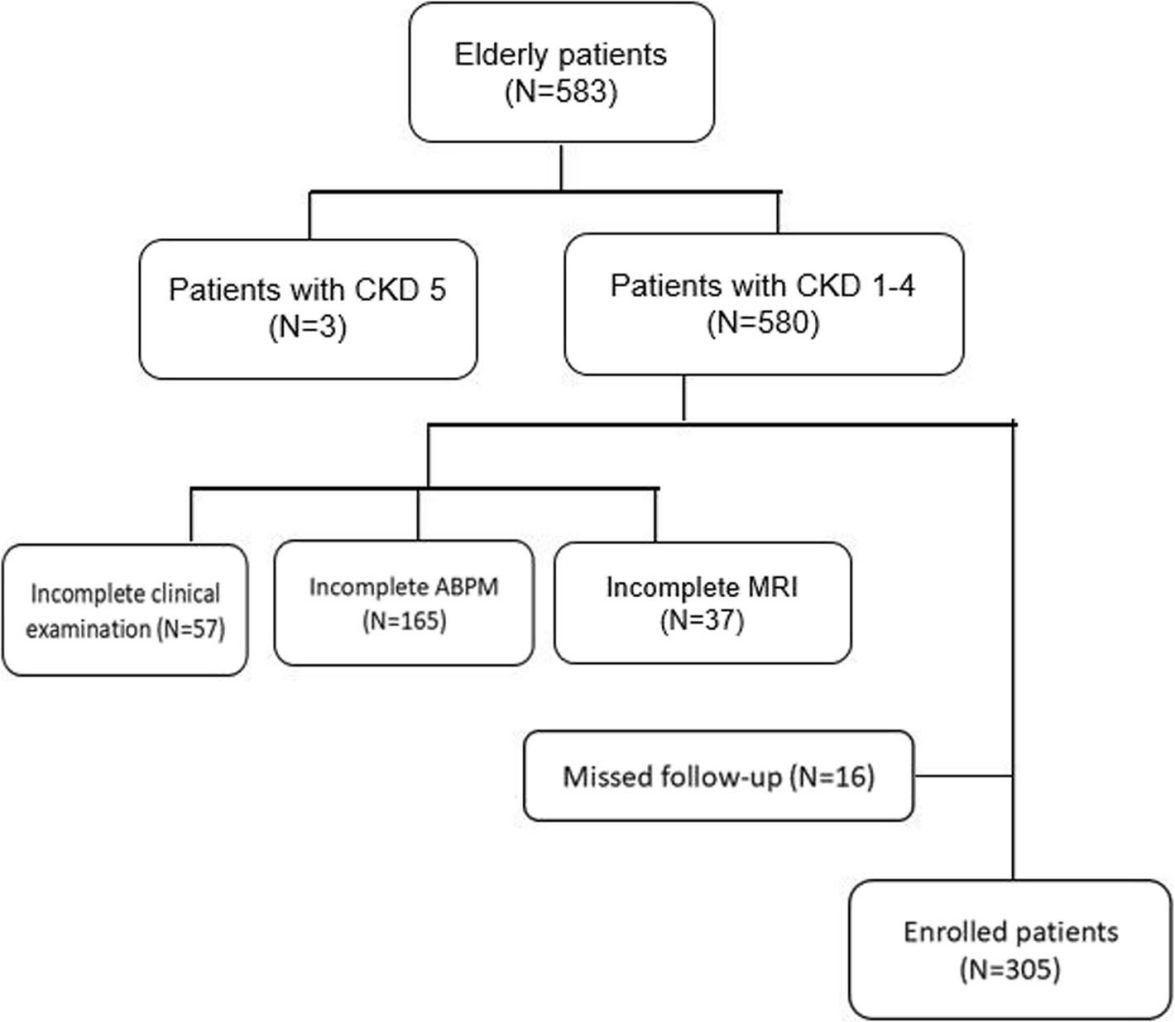
Flow diagram of the study design

This study included 583 inpatients, ≥65 years of age, who received cognitive assessment and cerebral MRI at the Geriatric Medicine department of the Beijing Friendship Hospital between January 2014 and September 2019. The participants were followed up until March 2020. Among these subjects, 3 were excluded for chronic kidney disease, 57 were excluded for incomplete clinical information, 165 were excluded for the incomplete ambulatory blood pressure monitoring (ABPM), 37 were excluded for the incomplete MRI, and 16 were excluded for missed follow-up. Finally, 305 participants were included in this analysis to assess the effect of BPV and its risk factors on cognitive function in elderly subjects (Fig. 1).

The study was approved by the ethics committee of Beijing Friendship Hospital, Capital Medical University (code: 2018-P2–120-01) and was conducted in accordance with the Declaration of Helsinki.

Demographic characteristics, including years of education, medical history, clinical information, and concurrent medications, were collected.

Hypertension was defined as systolic blood pressure (SBP) ≥140 mmHg, diastolic blood pressure (DBP) ≥90 mmHg, or ongoing therapy for hypertension. Type-2 diabetes was defined as glycosylated hemoglobin (HbA1c) ≥6.5%, a non-fasting plasma glucose concentration ≥ 200 mg/dL, fasting plasma glucose concentration ≥ 126 mg/dl, or if the patient was treated with oral hypoglycemic medications or insulin.

### Laboratory measurements

Lipids, including total cholesterol (TC), triglyceride (TG), low-density lipoprotein cholesterol (LDL-C), and high-density lipoprotein cholesterol (HDL-C), were measured using standard methods of the central laboratory of the Beijing Friendship Hospital. Fasting glucose, glycosylated hemoglobin (HbA1c), liver and renal function, and albumin (ALB) were also measured. The CKD-EPI (the Chronic Kidney Disease Epidemiology Collaboration equation) calculated the estimated glomerular filtration rate (EGFR).

### Ambulatory blood pressure monitoring

A validated ambulatory recorder (DMS-ABP, USA) and cuff were used on the non-dominant arm to perform ABPM. BP was measured at 30-min intervals for daytime (06.00–21.59 h) and 1-h intervals for night-time (22.00–05.59 h). BP, including 24-h mean value, mean daytime value, and mean night-time value, were calculated from recorded measurements and used in data analyses.

The nocturnal BP fall (%) was calculated using the following formula: (daytime SBP  − night-time SBP)/daytime SBP. We classified the nocturnal BP fall into the following three patterns: the dipper pattern if the nocturnal BP fall was > 10%; the non-dipper pattern, if the nocturnal BP fall was between 0 and 10%; and the rising pattern, if the nocturnal BP fall was < 0%. Patients with an extreme dipper pattern (nocturnal BP fall > 20%) were combined with those with the dipper pattern due to the limited number of cases (*n* = 6). Nocturnal hypertension was defined as a night-time SBP ≥120 mmHg and/or night-time DBP ≥70 mmHg, based on the 2014 guidelines for the management of hypertension published by The Japanese Society of Hypertension.

### Magnetic resonance imaging protocol and assessments

MRI images were acquired using a 3.0 T scanner (Siemens, Berlin, Germany) in the Radiology Department of our hospital. Sequences included T2-weighted imaging (T2WI), T1-weighted imaging (T1WI), diffusion-weighted imaging (DWI), fluid-attenuated inversion recovery imaging (FLAIR), and susceptibility-weighted imaging (SWI). The main parameters were as follows: repetition time (TR) = 4500 ms, echo time (TE) = 84 ms, flip angle (FA) = 120°, matrix = 256 *256, field of view (FOV) = 220 * 220 mm^2^, slice thickness = 5 mm, and slice gap = 1 mm, number of slices = 24.

White matter hyperintensities (WMHs) were identified as hyperintense areas in the periventricular and deep white matter on both T2WI and FLAIR. WMH was rated using the Fazekas scale on FLAIR images. Lacunar infarcts (LACs) were defined as small (diameter of 3–15 mm), sharply demarcated hyperintense lesions on T2WI with corresponding foci of FLAIR low signal intensity and assessed in the basal ganglia, internal capsule, thalamus, brainstem, radiating crown, and semioval center. The number of WMHs and LCIs were calculated to assess the severity of cerebral small vessel disease. MRI assessments were performed by two experienced neuroradiologists who were blinded to the clinical information.

### Measurement of cognition

Cognitive function was assessed by the Mini-Mental Status Examination (MMSE) with a total score 0–30. It included 11 items, categorized as: orientation (10 points), memory registration (3 points), attention and calculation (5 points), recall (3 points), language (10 points). A 27-point was proved to be an appropriate cutoff for cognitive impairment in a population with relatively high educational attainment [8].

Follow-up was performed by reviewing data from medical records or by telephone interviews. The primary outcome was all-cause mortality. Secondary endpoints were major adverse cardiac and cerebrovascular events (MACCE), including acute coronary syndrome (acute myocardial infarction or unstable angina pectoris), heart failure of NYHA III-IV, stroke/transient ischemic attacks (TIA), and cerebral hemorrhage.

### Statistical analysis

All continuous variables were reported as mean ± standard deviation (M ± SD), and categorical variables were presented as counts and percentages. The Kolmogorov–Smirnov test was used for the normality assumption. Pearson’s chi-squared test was used to investigate the categorical variables. For the analyses of continuous variables with normal distribution, a t-test was used to examine the differences between the normal cognition and impaired cognition patients. Kruskal–Wallis with post hoc Mann–Whitney U-test was used to analyze the continuous variables with non-normal distribution. Correlations between nocturnal BP fall and MMSE were evaluated using linear regression. Cox regression and Kaplan–Meier curves were used for survival analysis. All statistical analyses were performed using SPSS statistical software version 24.0 for Windows (SPSS Inc.), and a *p*-value < 0.05 was considered statistically significant.

## Results

### Baseline characteristics of participants

A total of 305 participants with a mean age of 81 years were included in the study. The average years of education received was 14; 69% of participants were men; the average BMI was 24; 10.2% were smokers; 79% had hypertension, and 39.3% had diabetes. As shown in Table 1, 35.7% of subjects were taking antiplatelet therapy, 46.5% were on a statin, 41.3% were on an ACEI or ARB, 43% were on a CCB, and 30.2% were taking beta-blockers. The average HbA1c was 6.1%. The average TC was 4.0 mmol/l, TG was 1.3 mmol/l, and LDL-C was 2.4 mmol/l.

**Table 1:** Demographic characteristics of the study population

	All subjects (*N* = 305)	NCI group (*n* = 175)	CI group (*n* = 130)	*p*-value
Age, years	80.6 ± 7.6	79.1 ± 7.3	82.5 ± 7.7	< 0.001*
Male, n (%)	210 (69)	125 (71.4)	85 (65.4)	0.260
Education, years	14.0 ± 3.6	14.8 ± 3.0	13.0 ± 4.0	< 0.001*
BMI, kg/m^2^	24.3 ± 3.6	24.5 ± 3.6	23.9 ± 3.6	0.150
Smoking, n (%)	31 (10.2)	17 (9.8)	14 (10.8)	0.124
Hypertension, n (%)	242 (79)	136 (77.7)	106 (81.5)	0.415
Diabetes, n (%)	120 (39.3)	67 (38.3)	53 (40.8)	0.661
FG, mmol/L	5.7 ± 1.8	5.5 ± 1.3	6.1 ± 2.3	0.016*
HgB	130.1 ± 15.7	132.0 ± 15.0	127.6 ± 16.3	0.015*
ALB	37.5 ± 4.5	38.5 ± 4.3	36.2 ± 4.5	< 0.001*
HbA1c, %	6.1 ± 1.6	5.9 ± 1.0	6.0 ± 1.1	0.168
Cr, mmol/l	75.5 (25.2–205.4)	73.5 (25.5–166.9)	79.8 (34.8–205.4)	0.021*
eGFR, ml/min/1.73m^2^	77.7 (17.3–113.9)	79.2 (32.6–113.9)	72.1 (17.3–101.8)	< 0.001*
TC, mmol/L	4.0 ± 1.1	4.0 ± 0.9	4.1 ± 1.3	0.541
TG, mg/dL	1.3 ± 0.8	1.3 ± 0.9	1.2 ± 0.7	0.638
HDL-C, mg/dL	1.2 ± 0.3	1.2 ± 0.3	1.1 ± 0.3	0.634
LDL-C, mg/dL	2.4 ± 0.8	2.4 ± 0.7	2.4 ± 1.0	0.478
Antiplatelet, n (%)	109 (35.7)	62 (35.4)	47 (36.2)	0.896
Statin, n (%)	151 (46.5)	93 (53.1)	58 (44.6)	0.141
ACEI/ARB, n (%)	126 (41.3)	72 (41.1)	54 (41.5)	0.945
CCB, n (%)	263 (43)	69 (39.4)	52 (40)	0.920
beta-blocker, n (%)	92 (30.2)	54 (30.9)	38 (29.2)	0.760
WMH	2 (0–3)	1 (0–3)	2 (0–3)	< 0.001*
LCI	1.64 ± 2.66	1.52 ± 0.71	1.88 ± 0.81	0.019*
MMSE	25.55 ± 4.81	28.66 ± 1.05	21.36 ± 4.72	< 0.001*

Values are mean ± SD, n (%), or median (interquartile range). *BMI* Body mass index, *FG* Fasting glucose, *HgB* Hemoglobin, *ALB* Albumin, *HbA1c* Hemoglobin A1c, *Cr* Creatinine, *eGFR* Estimated Glomerular Filtration Rate, *TC* Total cholesterol, *TG* Triglycerides, *HDL-C* High-density lipoprotein, *LDL-C* Low-density lipoprotein, *ACEI* Angiotensin-converting enzyme inhibitor, *ARB* Angiotensin receptor blocker, *CCB* Calcium channel blocker, *WMH* White matter hyperintensities, *LCI* Lacunar cerebral infarction, *MMSE* Mini-Mental Status Examination. *: *P* < 0.05.

Among 305 participants, 42.6% were identified as having CI with MMSE scores < 27. Subjects with (CI group, *n* = 130) and without CI (NCI group, *n* = 175) were compared according to demographic and clinical characteristics and risk factors. As shown in Table 1, compared to NCI subjects, subjects with CI were significantly older (82.5 ± 7.7 vs. 79.1 ± 7.3 years, *p* < 0.001), less educated (13.0 ± 4.0 vs. 14.8 ± 3.0 years, *p* < 0.001), had higher fasting glucose (6.1 ± 2.3 vs. 5.5 ± 1.3 mmol/l, *p* = 0.016), lower HgB (127.6 ± 16.3 vs. 132.0 ± 15.0 g/dl, *p* = 0.015), significantly lower ALB (36.2 ± 4.5 vs. 38.5 ± 4.3 g/dl, *p* < 0.001), significantly lower eGFR (72.1 vs. 79.2 ml/min/1.73m^2^, *p* < 0.001), and more severe WMH (2 vs. 1, *p* < 0.01) and LCI (1.88 ± 0.81 vs. 1.52 ± 0.71, *p* = 0.019).

After an average follow-up of 2.03 ± 1.45 years, there were 35 all-cause deaths. Among them, 21 patients died due to pneumonia or abdominal infection, 4 due to cancer, 4 due to cardiovascular disease, and 6 due to other diseases. A total of 33 cases suffered from MACCE. Among them, 21 patients had stroke/TIA or cerebral hemorrhage, while there were 12 cases of acute coronary syndrome or acute heart failure.

### BP variation and cognitive impairment

**Fig. 2:**
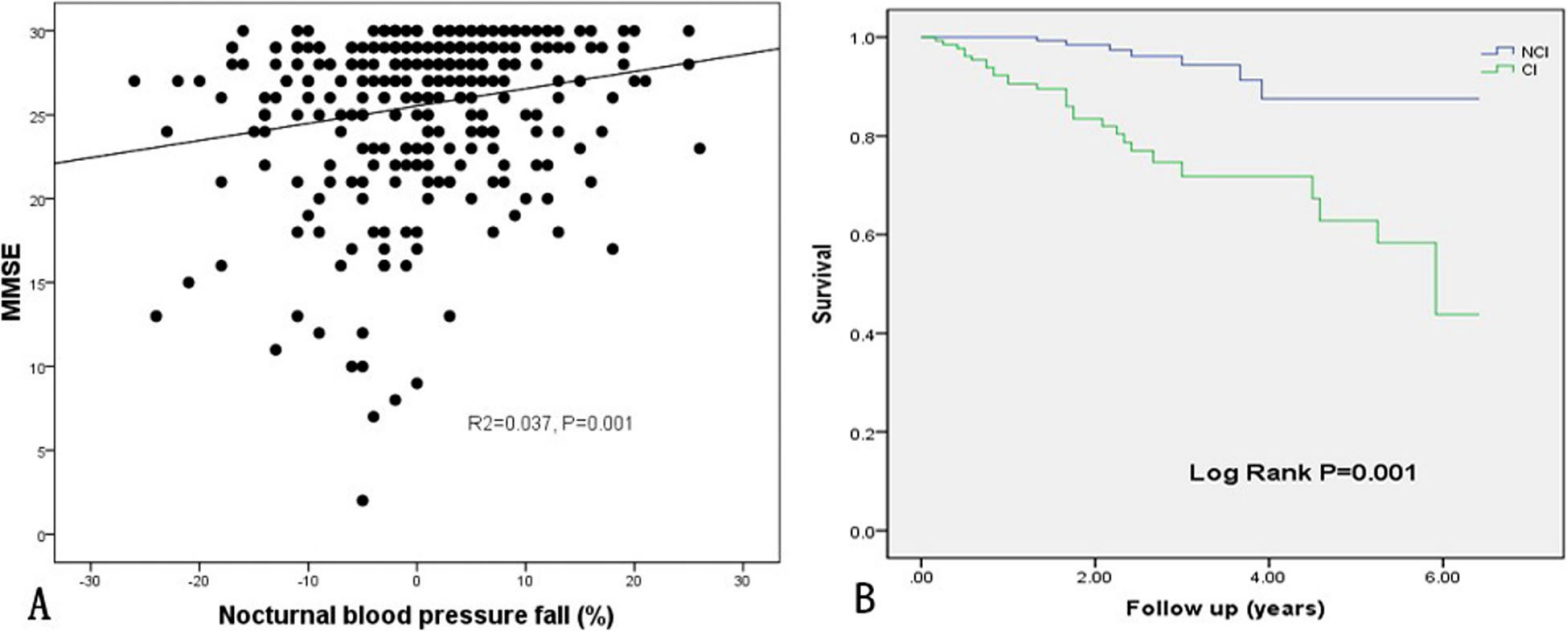
Linear regression and K-M curve. **A** linear regression between nocturnal BP fall and MMSE. **B** K-M curve of cognitive impairment on the all-cause mortality. Note: NCI, non-cognitive impairment; CI, cognitive impairment

BP was reasonably controlled, with an average SBP of 127 mmHg and DBP of 66 mmHg. According to ABPM, 13.1% of patients had a dipper pattern, 45.6% had nocturnal BP rise, and 41.3% had a non-dipper pattern. Compared with NCI patients, the patients with CI had significantly higher night-time SBP (130.0 ± 18.2 mmHg vs. 123.9 ± 15.1 mmHg, *p* = 0.002), and more patients had nocturnal BP rise (52.3% vs. 40.6%, *p* = 0.042). In contrast, there was no significant inter-group difference in DBP. The incidence of daytime SBP and nocturnal hypertension was higher in the patients with CI compared to those with normal cognition; the difference was not statistically significant (Table 2). Nocturnal BP rise had a positive correlation with MMSE (R^2^ = 0.037, *p* = 0.001) (Fig. 2A).

**Table 2:** Ambulatory blood pressure monitoring parameters according to the cognitive function

	All subjects (*n* = 305)	NCI (*n* = 175)	CI (*n* = 130)	*p*-value
Mean SBP, mmHg	127.4 ± 13.8	126.1 ± 12.5	129.2 ± 15.2	0.055
Mean DBP, mmHg	66 ± 8.5	65.9 ± 8.1	66.1 ± 9.0	0.887
dSBP, mmHg	127.1 ± 14.1	125.9 ± 13.2	128.7 ± 15.1	0.104
dDBP, mmHg	66.1 ± 8.7	66.1 ± 8.3	66.0 ± 9.1	0.908
nSBP, mmHg	126.5 ± 16.7	123.9 ± 15.1	130.0 ± 18.2	0.002*
nDBP, mmHg	64.4 ± 9.9	63.6 ± 9.3	65.5 ± 10.6	0.114
Nocturnal hypertension, n(%)	207 (67.9)	112 (64)	95 (73.1)	0.093
**BP category**				
Dipper, n(%)	40 (13.1)	24 (13.8)	16 (12.3)	0.719
Non-dipper, n(%)	126 (41.3)	80 (45.7)	46 (35.4)	0.070
Rise, n(%)	139 (45.6)	71 (40.6)	68 (52.3)	0.042*

*SBP* Systolic blood pressure, *DBP* Diastolic blood pressure, *CI* Cognitive impairment, *NCI* Non-cognitive impairment. *, *p* < 0.05.

According to the Cox regression models, CI was independently associated with all-cause mortality during long-term observation (95% CI, 6.001 (2.62–13.78), *p* < 0.001), with 500% increase during follow-up (Fig. 2B). Nocturnal BP rise had no significant predictive value for all-cause mortality in elderly patients.

## Discussion

Cognitive impairment (CI) is rampant among the elderly worldwide. This study enrolled 305 cases, with a mean SBP of 127.4 mmHg and DBP of 66 mmHg. Among them, 130 (42.6%) participants were identified with CI. These patients had worse nutrition and kidney function than individuals with normal cognition, which negatively affected their life quality and long-term prognosis. Hypertension is a major risk factor for cognitive dysfunction [11, 12], and the results of the Honolulu Asia Aging Study demonstrated a positive association between lower brain volume, neuritic plaques, and hypertension [12]. However, the relationship between BP variation and cognitive function remains unclear.

This study demonstrated that in elderly patients (mean age of 81 years), night-time SBP and nocturnal BP rise were positively correlated with CI, even if the patients had normal BP. Mean BP and daytime BP showed no correlation. Nocturnal hypertension was not significantly higher in the CI group. These results are consistent with the findings of Manabu Kokubo and his colleagues [11], who reported that higher night-time SBP levels contributed to greater WMH volumes in elderly hypertensive patients. High nocturnal BP is also associated with an increase in cardiovascular events [13] and the onset of chronic kidney disease [14] .

Despite enormous evidence supporting the role of atherosclerosis in the pathogenesis and progression of CI, the mechanistic relationship between nocturnal BP and CI remains unknown. Following are some of the possible mechanisms: first, nocturnal BP rise decreased cerebral blood flow and was related to higher levels of insulin resistance markers in normotensive and untreated mildly hypertensive adults [15]. Second, non-dipping of nocturnal BP increased the markers of endothelial dysfunction and inflammation, which are proposed as candidate mechanisms of atherosclerosis [16]. Moreover, cerebral small vessel disease, including LCI and WMH, had a major role in the senile vascular CI [2, 17]. This study also demonstrated that elderly patients with CI had more severe WMH and LCI than those without CI. Nocturnal BP rise contributed to greater WMH volumes but not LCI. This may be because WMH is more sensitive to the atherosclerotic risk factor than LCI.

We observed that nocturnal BP rise was associated with lower cognition, which could decrease MMSE by 2.9%. Although several studies have suggested that the impact of hypertension on cognition is global, not all studies broadly investigated the distinct cognitive domains. Some studies reported attention and executive functioning domains as cognitive domains that are most vulnerable to hypertension [18] since cognitive processes rely heavily on the integrity of frontal and subcortical brain structures, which may be most vulnerable to the effects of hypertension. These findings supported our hypothesis that the deterioration of orientation, attention, calculation, and language that occurs with aging is a BP variation-related impairment [19]. Some studies reported that processing speed is the first cognitive domain to be impacted by white matter lesion burdens due to uncontrolled hypertension. Jacobs et al revealed that WMH location influenced the relation between WMH and executive functioning and that parietal WMH is a significant contributor to executive decline in MCI [20]. This study did not assess the WMH location in detail.

The present study showed that baseline CI measured by MMSE score was associated with an elevated risk of all-cause mortality, which was consistent with previous studies [17, 18]. Yet, the prognostic value of the nocturnal BP rise has not been established. The Ambulatory Blood Pressure Collaboration in Patients With Hypertension (ABC-H) examined this issue in a meta-analysis of 17,312 hypertensive patients (mean age of 50–70 years) who were followed up for 4–8 years. They found that the non-dipping pattern could predict 33% of all-cause mortality and 57% of cardiovascular mortality after adjustment for 24-h SBP. Among the four different dipping subgroups, patients with nocturnal BP rise had the worst prognosis for cardiovascular events and all-cause mortality, and nocturnal BP rise was responsible for most of the non-dipping pattern adverse prognosis [19]. This study showed that the patients with nocturnal BP rise had a higher incidence of cardiovascular disease than those without nocturnal BP rise (15.1% vs. 7.2%, *p* = 0.027). Still, nocturnal BP rise was not significantly associated with all-cause mortality. Perhaps nocturnal BP rise was more closely associated with cardiovascular events than mortality. The patients in this study were much older, and most of them died of infection or another disease, while only 11.4% died of cardiovascular events. So it is important to emphasize the importance of controlling BP using ABPM as an indicator to control cardiovascular events [21].

The present study has some limitations. First, as the present study was a cross-sectional study, the cause-effect relationship was unclear. Second, in addition to LCI and WMH, cerebral microbleeds are also a form of cerebral small vessel disease, which has been reported to be highly prevalent in memory clinic patients and those with Alzheimer’s disease [22–24]. Third, the precise cause of cognitive impairment was not characterized in this study using molecular ligands or pathological confirmation.

## Conclusions

The present study showed that nocturnal BP rise contributed to cognitive impairment in elderly patients. CI increased the mortality in the elderly, but the nocturnal BP rise did not. Further studies are required to determine whether lowering nocturnal BP should be targeted to prevent the progression of CI.

## Data Availability

The datasets used during the current study are available from the corresponding author on reasonable request.

## Abbreviations

ABPM: Ambulatory blood pressure monitoring
MRI: Magnetic Resonance Imaging;
CI: Cognitive impairment
NCI: Non-cognitive impairment group
LCI: Lacunar infarcts
WMH: White matter hyperintensities
BP: Blood pressure
BPV: Blood pressure variation
SBP: Systolic blood pressure
DBP: Diastolic blood pressure
BMI: Body mass index
FG: Fasting glucose
HgB: Hemoglobin
ALB: Albumin
HbA1c: Hemoglobin A1c
Cr: Creatinine
eGFR: Estimated glomerular filtration rate
TC: Total cholesterol
TG: Triglycerides
HDL-C: high-density lipoprotein
LDL-C: Low-density lipoprotein
ACEI: Angiotensin-converting enzyme inhibitor
ARB: Angiotensin receptor blocker
CCB: Calcium channel blocker
MoCA: Montreal Cognitive Assessment
MMSE: Mini-Mental Status Examination
TIA: Transient ischemic attacks
